# Investigating the Use of Simulated Patient Ratings in Objective Structured Clinical Examinations to Enhance Undergraduate Medical Assessments in Riyadh, Saudi Arabia

**DOI:** 10.7759/cureus.65643

**Published:** 2024-07-29

**Authors:** Ahmed Mahmoud

**Affiliations:** 1 Family Medicine and Polyclinics, King Faisal Specialist Hospital and Research Centre, Riyadh, SAU

**Keywords:** undergraduate, objective structured clinical examinations, simulated patient, saudi arabia, domain-based, simulated patient (sp), assessment, osce

## Abstract

Background: Objective structured clinical examinations (OSCE) are the gold standard of clinical assessment, and are used to conduct undergraduate family medicine clinical assessment at King Faisal Specialist Hospital and Research Centre (KFSHRC). Some studies have suggested that simulated patient (SP) ratings could provide a better measure of empathy and communication skills than physician scores. The objective of this study is to further explore the effectiveness of simulated patient (SP) ratings in undergraduate OSCE assessments.

Methods: The research employed a mixed-method approach. Three OSCE assessments for final-year students were selected. Both physicians and SPs evaluated each student, providing global ratings across four domains. The quantitative aspect involved comparing physician and SP scores and assessing correlation. The qualitative aspect involved interviewing SPs to establish what student behaviours led to higher or lower scores.

Results: Moderate correlation was found between physician ratings and SP ratings (r=0.53, p<0.01). Internal consistency of the SP ratings was lower than physician scores. SPs considered themselves to be patient advocates and were keen to give formative feedback. The ability of the trainee to truly listen was a major concern. Scoring for SPs was relatively holistic in nature.

Conclusions: The results demonstrate that SP scores have slightly weaker reliability but are still relevant and offer a completely different perspective, enriching the assessment data. Assessment should take patient or SP perspectives into account, and not rely solely on the expert physician. Changing the assessment methods will lead to necessary changes in student approach to the OSCE and improve authenticity and validity.

## Introduction

Objective structured clinical examinations (OSCE) are standardised simulations designed to align with learning outcomes and curriculum objectives [1]. They are widely recognised as the benchmark for undergraduate medical assessment [2], with the assumption that OSCE performance correlates well with real-world clinical practice [3]. Research indicates a moderately strong correlation between OSCE scores and resident performance in clinical environments [4]. However, some researchers argue that OSCEs may not adequately measure certain essential skills and behaviors integral to the practice of medicine, which is both a science and an art [3]. These include communication skills, trainee attitudes, professionalism, teamwork and empathy, which are challenging to evaluate through OSCEs. O'Sullivan and Toohey conducted a review of multiple studies in Australia, discovering that a considerable number of iatrogenic injuries in Australian hospitals were more often attributed to lapses in professionalism rather than deficiencies in knowledge [5]. They concluded that students with proficient communication skills and professionalism are less likely to be involved in medical errors and are better equipped to handle such errors and their consequences openly and effectively.

Physician examiners are the ‘gold standard’ in assessing skills and behaviours in the OSCE format, but research has shown that there is great variability in the grades produced [6]. Examiners are susceptible to a wide range of biases including the halo effect, where a good first impression in the clinic leads to a more favourable OSCE score, or vice versa. Other biases include central tendency error, a tendency for the examiner to choose the middle ratings, and the hawk-dove effect, where some examiners are stringent and others lenient in their grading [7].

In this era of multi-source feedback and 360˚ feedback, patients are regularly asked to fill in patient satisfaction questionnaires as part of formative assessments of physician performance and annual appraisal. 360˚ assessment of trainees involves feedback from colleagues, trainers, patients, and other allied healthcare professionals as part of workplace-based assessment (WPBA). Patients should be considered experts at assessing professional consultation manner and effective communication [8]. Ultimately, it is the patient who is regularly exposed to a variety of doctors with different techniques, some more effective than others, putting the patient in a unique position to judge the effectiveness of the communication. In addition, it is the patient who has the most to lose from miscommunication in the consultation.

Communication skills are a vital component of the family medicine consultation. Poor communication skills are the most common cause of patient complaints [9]. Reliable assessment is a challenge as it will be intrinsically subjective. Studies support this assertion, showing lower inter-case and inter-rater reliability for assessing communication skills stations [10]. The National Board of Medical Examiners in the United States looked at ways to improve the assessment of communication skills in the licensing exam (United States Medical Licensing Examination) and they found that biomedical content was dominant in the assessment, resulting in trainees who were much more focused on gathering accurate, detailed information and planning suitable management than demonstrating a patient-centred consultation style. The Board suggested that scenarios should be based on real patients, and simulated patients (SPs) given more flexibility in their role, within reason, to improve authenticity [11].

Since the 1960s, trained SPs have been engaged in the training and assessment of healthcare providers [12]. Often, SPs are asked to provide formal feedback on their patient experience. There remains concern that SPs do not have sufficient training or experience to provide valid or reliable assessments of trainees. Jefferies et al. [13] found that SP overall ratings showed a weak but significant correlation with expert examiner ratings. However, SPs were more likely to give trainees the benefit of the doubt, perhaps due to limited knowledge and experience in the speciality. Additionally, the question remains whether SPs can distinguish between deep knowledge and more superficial surface knowledge. Berg et al. [14] found a strong positive correlation between trainee self-reported ratings and SP global ratings in the assessment of empathy, suggesting that trained SPs can provide meaningful assessment scores and feedback for use in a formative capacity, although evidence for summative use is still lacking.

The diversity and subjective experiences of SPs could add richness to the rating process, introducing a new dimension to the assessment, but the utility and reliability of this new dimension are still not fully understood. SP ratings might help us to capture more nuanced elements of competence and expertise. Blatt et al. [15] looked at “off the record” concerns that SPs had about undergraduate medical students during OSCE and concluded that these concerns might be valuable in identifying areas that needed improvement for individual students, as well as identifying curriculum needs if similar issues were flagged up for multiple students. Most of these concerns raised by the SPs were not identified when using the traditional assessment format [15].

Wilkinson and Fontaine [16] found that even untrained patients may be able to provide a valid, reliable score that contributes to a trainee’s assessment in both a formative and summative manner, with data illustrating a good correlation between patient scores and final summative OSCE grade. Sullivan et al. [17] found that SPs can support learner reflection in a formative capacity, through post-scenario feedback. In addition, the SP background, in terms of education level and experience in the healthcare field, was found to be a factor in the extent of the feedback that could be provided. The SP role could be developed further with continuous training and support. High quality training prepares SPs for competent role portrayal, as well as giving relevant feedback and accurate completion of assessment instruments [18].

SPs are one-third of the OSCE triad which also comprises the expert clinician and the trainee. They have their own unique insights and perspectives on proceedings, as they actively participate in the assessment process. Previous qualitative studies have suggested that SPs see themselves in the unique role of expert patients, and offer holistic assessments which cannot effectively be deconstructed [19]. Besides this, however, there is a lack of data in the literature on how SPs rate trainees, and how they decide on their scores.

This study aims to evaluate the effectiveness of SP ratings in the context of undergraduate OSCEs in our institution. The research seeks to identify the specific domains where SPs can provide valuable feedback, compare SP rating scores with those given by physicians, and determine if these SP ratings could be integrated into formal assessments.

## Materials and methods

Sampling strategy

At the end of their fifth-year Family Medicine clerkship, undergraduates complete four separate 10-minute OSCE stations with SPs and four different family physician evaluators. The study involved final-year students participating in the Family Medicine clerkship OSCE from October to December 2022 at the King Faisal Specialist Hospital and Research Centre, Riyadh. This included three cohorts of students, with 22-27 students per exam, each attending a four-station OSCE. In each station, a physician examiner assessed the OSCE using domain-based ratings (Appendix 1) organized into four domains, namely knowledge, clinical competence, empathy and communication skills. The examiners observed and evaluated the consultation silently. The SP also provided a domain-based score, divided into four similar domains (Appendix 2), namely communication skills, empathy, knowledge and level of confidence in the student. Even for SPs, Regehr et al. [20] found that global ratings were more reliable than checklist ratings. This would support the use of global or domain-based ratings for SPs. Prior to the commencement of the study, training sessions were organised in domain scoring for both the examiners and the SPs (Appendices 3-4).

Qualitative data collection

All SPs provided written consent to being interviewed for the study (Appendices 5-6). Semi-structured interviews were conducted for all the SPs involved in these three OSCE exams. A total of six SPs were recruited. All SPs were interviewed in private one-to-one interviews. For the qualitative data collection, interviews were chosen rather than focus groups or questionnaires. The objective was to elicit all their detailed ideas, concerns and opinions. Some of the required information may be quite personal and therefore sensitive. This might only be volunteered in a private one-to-one setting. An interview guide was formulated to help elicit the relevant data during the SP interviews (Appendix 7). Unfortunately, the small size of the sample meant that thematic saturation might be more difficult to achieve.

Quantitative data analysis

Quantitative data analysis began with calculating descriptive statistics for each scoring method. Then, the correlation between the datasets was examined. Specifically, physician and SP ratings were correlated within the same OSCE stations. Given that the data did not follow a normal distribution, a non-parametric test was required. Spearman's rank correlation was therefore utilized. Additionally, interstation reliability for each rating method was evaluated using Cronbach's alpha. This helped to gauge the internal consistency or reliability of the overall OSCE exam for each scoring method.

Statistical analysis was all done with the computer software program SPSS version 29 (IBM Corp., Armonk, NY).

## Results

Quantitative data

A total of 72 Al-Faisal University final-year medical students were included in this research. Each OSCE round comprised four separate OSCE stations. Each station was rated by domain ratings by the physician examiner and the SP. In the first round of the OSCE, 21 students participated; in the second round, 25 students participated; and in the third round, 26 students participated. No students declined or withdrew from the study, resulting in all 72 students completing the four OSCE stations.

**Figure 1:**
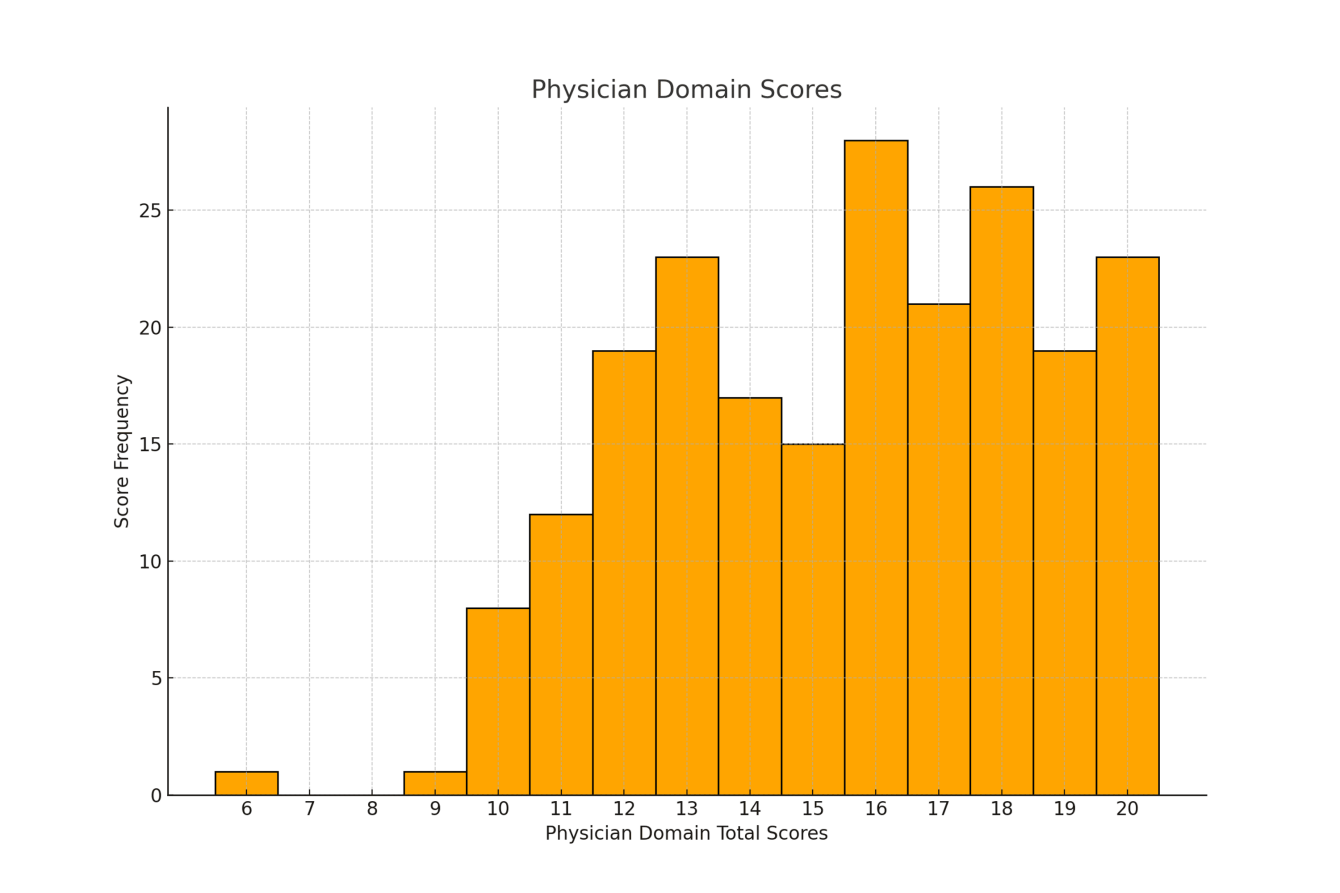
Distribution of physician domain scores The histogram shows the frequency of total scores (out of 20) in the physician domain scoring method

**Figure 2:**
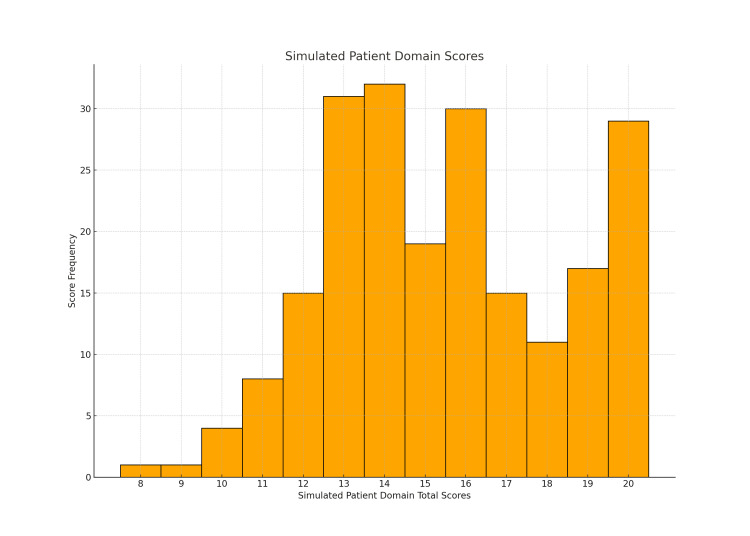
Distribution of SP total scores The histogram shows the frequency of total scores (out of 20) in the SP domain scoring method SP: simulated patient

Figure 1 displays the distribution of physician domain-based ratings across all three OSCE cohorts. Figure 2 illustrates the distribution of scores given by the SPs. The histograms indicate that, for both scoring methods, the OSCE scores did not follow a normal distribution. This observation was confirmed using the Shapiro-Wilk test in SPSS. The results of the test for each OSCE station in all three OSCE rounds revealed p-values of less than 0.05, meaning none of the OSCE station scores were normally distributed. 

Table 1 presents the quantitative data collected from the OSCEs. Each domain in the SP and physician ratings was scored out of 5, culminating in a total score of 20. These scores were then converted into percentages. The range of scores as well as the average, maximum and minimum total scores achieved by any student are highlighted for each of the two methods and for each specific domain. 

**Table 1:** Mean and range of scores across various ratings The data has been represented as percentage (%) scores in OSCE domain scoring for physicians and SPs OSCE: objective structured clinical examination; SP: simulated patient

Rating	Maximum (%) Student Score	Minimum (%) Student Score	Mean (%) Student Score
Physician domain - total	100	30	77
Physician domain - communication skills	100	20	76.5
Physician domain - knowledge	100	40	80.8
Physician domain - empathy	100	20	73.7
Physician domain - competence	100	40	77.2
SP domain - total	100	40	77.7
SP domain - communication skills	100	40	79.2
SP domain - empathy	100	40	74.6
SP domain - confidence	100	40	75.6
SP domain - knowledge	100	40	81.2

Looking at the mean scores (Table 1) for the whole group, the mean physician domain-based total score was 15.4 out of 20 (77%), and the mean SP total score was 15.5 out of 20 (77.7%). The minimum scores in the physician and SP domains were 6/20 (30%) and 8/20 (40%), respectively. 

Table 2 presents the Cronbach's alpha scores for each rating methodology across the different cohorts. These scores represent the internal consistency of the scoring methodology across the OSCE stations for each rating method in each cohort. A high Cronbach's alpha indicates that students' scores are consistent across all OSCE stations. Conversely, a low score suggests inconsistent scoring, which could imply issues with the overall exam, a particular station, or indeed the scoring method itself. Alternatively, it may indicate that the assessment is complex and multidimensional, measuring a variety of unrelated traits and skills.

**Table 2:** Cronbach’s alpha for the highlighted variable in each OSCE group The table shows the Cronbach's alpha coefficient obtained from the four-station OSCE exam for each cohort. OSCE: objective structured clinical examination; SP: simulated patient

Variables	Cohort 1	Cohort 2	Cohort 3
Physician domain - total	0.765	0.461	0.729
SP - total	0.501	0.518	0.664

Table 3 displays the Spearman's rank correlation between the different scoring methodologies. It shows the correlation between the total physician domain-based scores and the total SP scores for each cohort of students. Additionally, the correlations were calculated for the whole study population of 72 final-year students.

**Table 3:** Spearman's rank correlation coefficients for highlighted correlations The data is represented as Spearman's rank correlations between physician and SP domain OSCE scores for each cohort and the total group. Values are significant at p<0.01 level. OSCE: objective structured clinical examination; SP: simulated patient

Correlation	Cohort 1	Cohort 2	Cohort 3	All Cohorts
Physician domain - total vs SP domain - total	0.508	0.637	0.411	0.530

When comparing physician domain-based scores with SP scores, there was a moderately strong correlation, with a Spearman's coefficient of 0.530 (significant at the 0.01 level).

Qualitative data (SP interviews)

Six SPs were interviewed in English for the qualitative aspect of this study. There were three males and three females. All participants were between the ages of 30 and 50 years, and they were all fluent in the English language. All were employees of the hospital, three senior nurses, and three experienced administrative assistants. The SPs included two Saudi nationals, a British citizen and three Filipino expatriates. SP scores can be influenced by rater characteristics including gender, age, and healthcare experience, highlighting the importance of considering SP demographics when interpreting results [21].

Thematic analysis of the qualitative interview data was carried out according to the six steps set out by Braun and Clarke [22].

Patient Advocacy

One overriding theme that came through from the interviews was that the SPs took pride in the fact that they were involved in the assessment of students and were grateful that they were being given a voice and that their feedback was being valued. They saw themselves as representatives or advocates for the patients of these future physicians and felt a responsibility to make sure that patients' needs were met. 

One SP summed up the entire theme with the following quote: “I feel it’s so important that the patient perspective is being recognized in the assessment of future doctors.” Similarly, one SP noted “I wish that I could give this feedback to him. Maybe it would make him think more carefully about how he interacts.” Another SP summed it up concisely. “It’s so important that these students get not only the point of view of the doctor examiner, but also the point of view of the patient. We have a different point of view, and maybe different priorities too.”

Another linked theme that came through the interviews was regarding the overall score that was given to the student. The SPs were essentially scoring the students on the likelihood that they, as a real patient, would go back to this same student as a real physician for a real-life consultation. One SP mentioned, “I asked myself how comfortable I was with the student. If I was sick, would I trust them to treat me? I had that question in the forefront of my mind throughout.” This theme linked nicely into the first theme, with SPs advocating for patients, while grading essentially from a patient perspective. Another SP put it this way, “Maybe she knew her stuff, but as a patient I wouldn’t have wanted to go back to her, and of course that affected the grade she got from me.” The SPs were quite comfortable scoring the students’ empathy and communication skills, but they were not as comfortable scoring the level of knowledge, instead focusing on holistic judgements of all aspects of the consultation. One SP remarked, “It was difficult to assess their knowledge because I felt that I might be conned by a confident student. I was very aware of whether they had covered all the parts of the consultation that were important to me, like listening to my concerns, and involving me in making a clear management plan which made sense to me.” From the SP comments, the structure of the consultation was not an issue if the student was perceived to have covered all the relevant areas satisfactorily.

Benchmarking

It appeared that all the SPs were making judgements of the students based on their previous experiences of doctor-patient interactions, good and bad. Their expectations were shaped by previous interactions with physicians which were considered positive or professional, as well as bad experiences they were hoping not to revisit. Sometimes they would compare the student consultation to a previous doctor-patient interaction which they were reminded of. Usually, this was in a negative context but there were also positive comparisons made to "model" physicians. One SP remarked, "I remember seeing a physician back home who treated me in a similar way. Of course, I never went back." The expectation was that these students would behave as physicians, a construct in the mind of the SPs, built upon previous experiences and generally perceived societal expectations. Another SP remarked, "Why was he wearing scrubs and trainers? I couldn’t take him seriously. Family doctors don’t dress like that." Clearly, individual expectations were very important. Different SPs had different priorities when assessing the students’ performances. While some SPs might have given appearance special importance, others were looking for a doctor who exuded confidence and would instil confidence in them. "If they are confident and persuasive in what they are delivering, like a real doctor, then I’ll buy it. If they’re convinced, I will be convinced."

The Power of Listening

In terms of the grading of the students, this was the overarching theme. SPs felt that this was the key to a successful consultation. All the SPs agreed that a common problem was that they were not being adequately listened to. One SP stated, “They talk to you for the sake of getting information. It’s not a real conversation. They just say what’s in their heads, as if they’re speaking to themselves. They don’t see me!” Another noted, “If they interact well with me, listening and responding to what I say, like a real conversation, then I would score them highly. I need to feel listened to.” The theme was summed up by one SP when they said, “Many weren’t listening. That’s how I felt. When they were asking about concerns and expectations, they rarely let me finish. It seemed like a checklist exercise for them. Like a charade for the examiner. Some of them didn’t even bother to wait for my answers.” The above quotes were instructive in that it was clear that the perception of being listened to was vital to the confidence of the SP and was the starting point for a successful consultation. As a follow-on from this theme, body language was also noted to be important by all SPs, including eye contact, tone of voice, warmth and smiling. “Eye contact was so important for me. Looking down and fidgeting was a real no-no.” Similarly, another SP mentioned, “Tone of voice was critical for me. Some students sounded tired, some bored and monotonous, others were tense, which made me tense. The students who were calm but upbeat and engaged, they’re the ones I gave better grades to.”

Little Things Matter

The final theme which I was able to extract from these interviews was the importance that SPs attached to things that students might perceive as inconsequential. For example, one SP stated, “One student checked back on my responses, like he was double-checking. It really increased my confidence in him, and the grade too.” Another mentioned, “One student lowered their chair to be at my level. That immediately made me feel more positive about them and the whole consultation.” On a negative note, one SP commented, “She cut me off as I was trying to give her relevant information. As a patient that really hurt. I don’t think she realized how much something like that affects the whole experience.”

Table 4 provides a summary of the main themes and sub-themes extracted from the analysis of the SP interviews.

**Table 4:** Main qualitative themes that arose from the qualitative simulated patient interviews and example narratives Main themes and sub-themes extracted from the qualitative simulated patient interviews and example quotes

Sub-Theme	Example Narrative	Main Theme Category
Desire to give feedback	“I wish that I could give this feedback to him. Maybe it would make him think more carefully about how he interacts.”	Patient advocacy
Importance of patient perspective	“I feel it’s so important that the patient perspective is being recognized in the assessment of future doctors.”	Patient advocacy
Level of comfort with student	“I asked myself how comfortable I was with the student. If I was sick, would I trust them to treat me? I had that question in the forefront of my mind throughout.”	Benchmarking
Holistic judgements	“It was difficult to assess their knowledge because I felt that I might be conned by a confident student. I was very aware of whether they had covered all the parts of the consultation that were important to me, like listening to my concerns, and involving me in making a clear management plan which made sense to me.”	Global assessment
Comparison to previous experiences	“I remember seeing a physician back home who treated me in a similar way. Of course, I never went back.”	Benchmarking
Long-held expectations	“Why was he wearing scrubs and trainers? I couldn’t take him seriously. Family doctors don’t dress like that.”	Benchmarking
Not being heard	“They talk to you for the sake of getting information. It’s not a real conversation. They just say what’s in their heads, as if they’re speaking to themselves. They don’t see me!”	The power of listening
Body language	“Eye contact was so important for me. Looking down and fidgeting was a real no-no.”	The power of listening
Tone of voice	“Tone of voice was critical for me. Some students sounded tired, some bored and monotonous, others were tense, which made me tense. The students who were calm but upbeat and engaged, they’re the ones I gave better grades to.”	The power of listening
Checking back	“One student checked back on my responses, like he was double-checking. It really increased my confidence in him, and the grade too.”	Little things matter
Real engagement	“One student lowered their chair to be at my level. That immediately made me feel more positive about them and the whole consultation.”	Little things matter

Summary

The findings would suggest that once the SPs are immersed in the OSCE experience, they put themselves in the patient's shoes and become advocates of the patient, feeling at the same time pride and a responsibility to ensure that the trainee is providing an adequate level of care and will provide a reasonable level of care to their patients in the future. Furthermore, SPs judge the students mainly based on their previous experiences and they compare the trainee's performance to good and bad previous physician encounters. SPs seemed to provide a more holistic grade to students, more dependent on the feel of the whole consultation than any specific aspects. An important question was the likelihood that they would return to this physician if they were sick. How comfortable did they make the SP feel? The most crucial component of this comfort was whether the SP felt listened to. Were they asking questions solely for the examiner, or were they listening for answers and asking follow-up questions? Other aspects of body language were also seen as very important in the consultation, as were small kindnesses and gestures which showed that the student was engaged and that they cared. In contrast, it seemed that knowledge and clinical skills were not issues that were open to assessment by SPs, although they did respond to students who appeared calm and self-confident.

## Discussion

When comparing SP scores to physician scores, the overall mean scores were very similar, with moderate positive correlation, suggesting a reasonable level of agreement in the scoring between these two holistic scoring methods. SPs did, however, seem reluctant to give the lowest scores for any domains. The lowest ratings given by SPs were 2/5 or 40%, whereas physicians saw fit to give 1/5 to perceived poor performers. This is in keeping with the conclusions of McLaughlin et al. [23], who found that SPs are more likely to give trainees the benefit of the doubt.

Particularly in family medicine, the cornerstone of practice is the doctor-patient relationship or partnership, which encompasses patient-centred care, eliciting any hidden patient agendas and embarking on a shared management plan. The consultation is more akin to a shared journey than a doctor-dominated interaction. It naturally must follow that as patients are more involved in the consultation and more involved in their own management, they must also be more involved in quality management and clinician skills development. Patient feedback or patient satisfaction is now commonly part of physician appraisal, but the same cannot be said about trainee assessments, where the patient perspective seems to be somewhat ignored. The analysis of SP interviews suggests that SPs, acting as patient advocates, are more than happy to be involved in trainee assessment, both in a summative, and even more in a formative capacity. They are proud to represent patients and keen to give feedback to the trainees themselves. SPs give general, holistic assessments, benchmarked against previous personal experiences of consultations. They were not comfortable deconstructing the holistic grade into domains, as for them the assessment was based on the whole package. The necessary traits, skills and behaviours were too interlinked for the SPs to be able to separate the assessment into distinct strands.

Some behaviours were particularly important to the SPs. Top of the list was the feeling that they were being listened to and that the trainee was not just enacting an elaborate charade for the purpose of the examiner. The SP needed to be seen and heard, and for their prompts to be picked up on with relevant follow-up questions. Body language was vital with eye contact, tone of voice and warmth all seen as powerful positives. Even minor gestures, which might not be noticed by trainees or even examiners, could significantly affect the grades given. 

In a specialty that emphasizes relationship-building and patient-centered consultations, it is essential to incorporate a diverse range of evaluation information. This approach enhances the assessment process, leading to the creation of more holistic and personalized evaluation data. Such comprehensive assessments can be used both formatively and summatively to improve patient care. There might even be an argument for separate progression criteria, whereby a trainee who performs particularly badly in any of the scoring systems, including SP ratings by well-trained SPs, should not be allowed to proceed without remedial measures. Alternatively, some kind of ‘yellow card’ system could be implemented, whereby trainees would receive feedback regarding any specific concerns raised by SPs, and depending on the gravity of the concern, they may again be required to go through a remedial process [24].

Schuwirth and van der Vleuten [25] also found that trained SPs scored trainees with moderately high reliability. The findings support the inclusion of SP markers in assessments, a much-needed development in this age of the 360˚ feedback. This provides an opportunity to consider different assessors with varied perspectives, each one valuable, adding richness to the understanding of the student’s performance. Qureshi and Zehra [26], found that SPs can offer formative feedback to students which can lead to positive enhancement of trainee communication skills. The SPs saw a lot of potential in the OSCE as a formative assessment, whereby feedback could directly contribute to the development of the trainee and the uncovering of blind spots in their consultation manner which even the physician experts were unable to unearth, most likely because they were not really looking for these issues. This could offer a profound opportunity for trainees to benefit from a totally different perspective. The upshot of the SP feedback in the interviews suggests that far more needs to be done in terms of communication and body language, with the ability to truly listen to patients occupying the most powerful position in the trainee shortcomings. Incorporating SP ratings into assessments not only promotes the engagement of SPs in the assessment process, but also sends a clear message to the trainees that holistic patient-centred care is a crucial ingredient of a successful consultation. The natural knock-on effect would be a greater trainee focus on communication skills, a welcome development.

The patient perspective must be represented, even in undergraduate exams, and SP scores provide an excellent means of incorporating this information. Currently, SP appraisal should focus on communication and people skills, rather than knowledge and clinical competence. Clearly, SPs would need to go through a training process with relevant rubric, to aid standardisation of the scoring across the various stations. Well-trained SPs might also be given more leeway during the consultation, to interact more naturally and authentically.

The judgement of competence must not be purely doctor-driven, as this could potentially lead to proliferation and affirmation of unhelpful attitudes which are entrenched within the culture of the profession [3]. The "hidden curriculum" describes the manner in which professional norms and values may be unknowingly transmitted to future physicians, sometimes even undermining formal messages [27]. These ideas are passed to the trainee by example, on a day to day basis, and may include extremely negative traits, including ethical erosion, and the erosion of empathy. Accordingly, judging competence should not be limited to physicians from within the same professional culture. Giving patients a key role to play in the assessment and development of future physicians might help these young doctors become more aware of the importance of a patient-centred approach to practice, compelling them to focus more on empathic consultations, open and sincere body language, and the art of listening.

Further studies also need to be done on the effect of direct formative feedback to trainees from patients and SPs, as the SPs were, in general, very keen to be heard in a formative capacity, while reminders directly from them might be more powerful than suggestions from physician colleagues. SP ratings encompass areas that physicians are not trained to accurately assess, which might be considered blind spots due to the culture of assessment and the vantage point and main focus of the physician expert. Viewing the consultation through the SP lens adds a fresh perspective and provides a more holistic view of trainee consultation skills. In this era of continuous professional development, healthcare professionals are regularly appraised by peers and patients. It is only natural therefore that clinical assessment should involve assessment components from experts as well as the patient experience. 

SP ratings are constructed upon the individual experience of the SP from multiple previous healthcare encounters. Through these encounters, the SPs have built certain expectations for all aspects of the consultation. It is true that these expectations are unique to each individual, built upon their attributes, previous experiences and other environmental factors, but that does not make their appraisal of the trainee any less valuable or relevant. Indeed, Homer and Pell [28] found that addition of SP scores to physician checklist scores actually improved the reliability of the assessment, which may seem counter-intuitive in view of the subjectivity of their ratings. Regardless of the subjectivity, it might be that effective SP training with clear grading rubric, as well as the generally accepted norms for successful consultations among patients, result in these seemingly subjective ratings improving the objectivity and overall quality of the assessment. 

Meanwhile, physicians often have a completely different personal experience of being a patient, often consulting colleagues or friends coupled with the fact that their medical background would bring different factors into play during any consultation with a healthcare provider. Unlike our patient experts (the SPs), physician experts are possibly not in the best position to understand the perspective of the average patient consulting their family doctor. Currently, the limited evidence available suggests that doctors are reluctant to seek medical care. Gerada [29] found that family physicians generally resort to corridor consultations with professional colleagues or self-care. The old adage that doctors make the worst patients, might then possibly also be extended to the supposition that doctors may well not be best placed to truly understand the perspective of the patient in the classic doctor-patient consultation, or the typical patient experience. 

Possible limitations include the fact that this study is a single-centre study, carried out in one specific institution. Trainees were all final-year undergraduates from the same university in the same location. This could imply limited generalizability or transferability of the study findings. Additionally, the SPs had varied roles within the hospital, such as secretarial and nursing staff, with different levels of experience. Despite all attending a training session before the OSCE, their grading methods may have differed significantly. A further limitation of this study was the relatively low number (six) of SPs recruited. This was unavoidable as the OSCE exam was a four-station exam and the same group of trained SPs were often present in each exam. Clearly, this reduces the likelihood of reaching thematic saturation.

## Conclusions

Assessment drives learning, and students will generally learn what they need to learn and act how they need to act to succeed in their chosen field. If assessment designers see traits or skills as important, then that must be reflected in the assessments. The concerns of the SPs were a real eye-opener, which suggest that despite the coaching in patient-centred care and various communication skills sessions, if these were not adequately represented in the assessment then they would be largely ignored. The incorporation of SP perspectives might go some way to rectifying this, and aid students to cultivate helpful consultation traits and skills which will manifest themselves in their day-to-day practice.

## Figures and Tables

**Figure 3:**
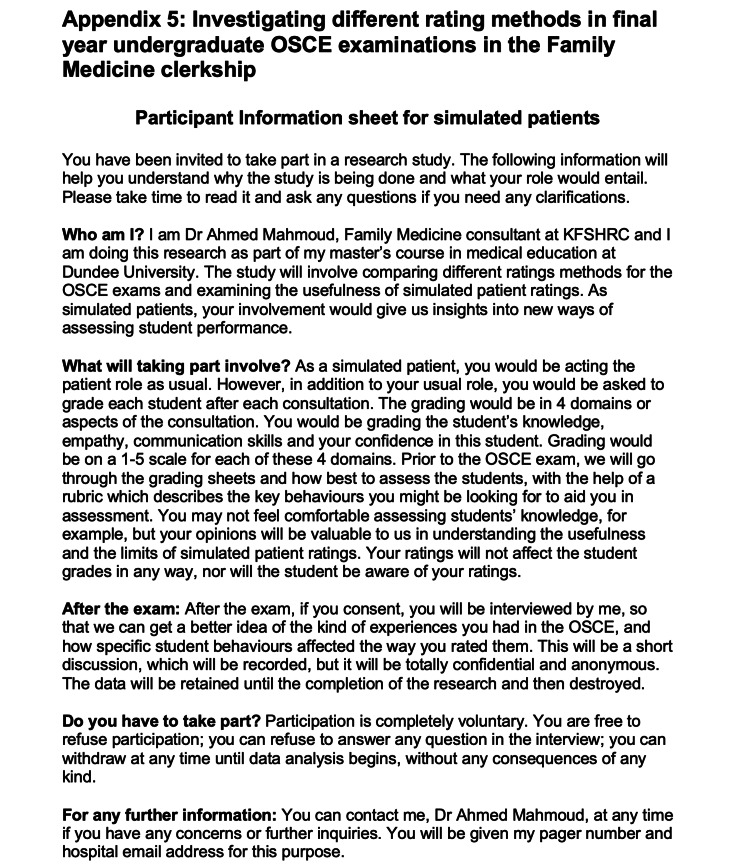
Appendix 5 (Participant Information sheet)

**Figure 4:**
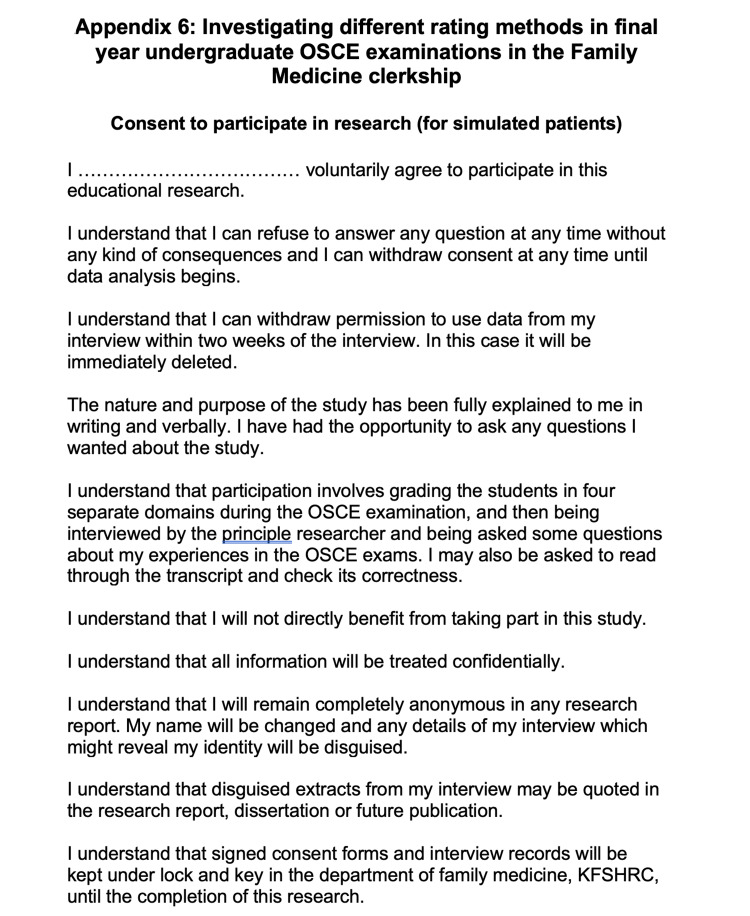
Appendix 6 (Consent Form Page 1)

**Figure 5:**
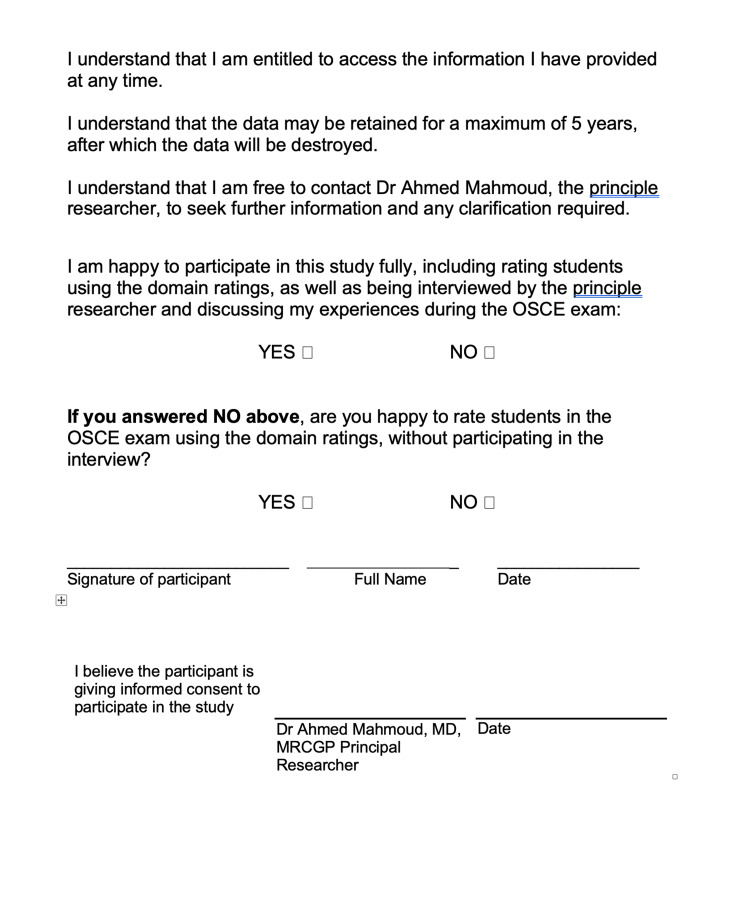
Appendix 6 (Consent Form Page 2)

## Appendices

Tables 5-8 show the marking sheets for the physician examiners and simulated patients.

**Table 5:** Appendix 1 (Examiners marking sheet) Domain-based marking sheet for Physician Examiners

Appendix 1: Domain-based marking sheet for Physician Examiners (out of 20) Circle appropriate score for each of the 4 domains below:				
Knowledge				
Very Poor	Poor	Average	Good	Very Good
1	2	3	4	5
Communication Skills				
Very Poor	Poor	Average	Good	Very Good
1	2	3	4	5
Empathy				
Very Poor	Poor	Average	Good	Very Good
1	2	3	4	5
Clinical Competence				
Very Poor	Poor	Average	Good	Very Good
1	2	3	4	5

**Table 6:** Appendix 2 (Simulated Patient Marking Sheet) Domain-based marking sheet for Simulated Patients

Appendix 2: Domain-based marking sheet for Simulated Patients (out of 20) Circle appropriate score for each of the 4 domains below:				
Knowledge				
Very Poor	Poor	Average	Good	Very Good
1	2	3	4	5
Communication Skills				
Very Poor	Poor	Average	Good	Very Good
1	2	3	4	5
Empathy				
Very Poor	Poor	Average	Good	Very Good
1	2	3	4	5
Confidence in this student				
Very Poor	Poor	Average	Good	Very Good
1	2	3	4	5

Tables 7-8 show the physician examiner and simulated patient rubrics. 

**Table 7:** Appendix 3: Rubric for Simulated Patients – How to assess students in OSCE domains [30] Rubric used for training simulated patients for domain OSCE ratings [30]

	1 – Very Poor	2 – Poor	3 - Average	4 – Good	5 – Very good
Empathy	Little or no empathy. No consideration to the needs of the patient. Self-involved, egocentric. No response to cues or concerns.	Understands that the patient has needs. Little response to patient needs. Limited perspectives. Brushes over patient concerns.	More connected to how the patient sees things and patient perspectives. Makes an attempt to address patient concerns.	Good connection to patient. Better attempt to address patient’s ideas, concerns and expectations. Patient’s thoughts and feelings are being addressed.	Able to put themselves in the patient’s shoes. Addresses patient’s ideas and concerns and actively responds. Patient feels completely at ease.
Communication skills	Closed body language. Closed leading questions. Looking at paper or monitor. No eye contact. No rapport established. Patient not at ease. No information shared. Uses a lot of Medical jargon	Tense body language. Turns slightly to speaker. Minimal eye contact. Mostly closed questions. Concentrated on gathering info. Use of medical jargon	More relaxed. Intermittent eye contact. Turned more towards the patient. Some open questions. No significant response to patient concerns – passive listening.	Mirrors body language of patient. Sustained eye contact. Mixture of open and closed questions. Good rapport. Makes an attempt to share information with the patient. Minimal medical jargon.	Relaxed and open. Natural eye-contact. Listens attentively. Shows interest and respect. Starts with open questions and allows patient to express themselves. Active listening. Picks up cues. Puts patient at ease. Shares and checks understanding and management plan. No medical jargon.
Knowledge	Not at all convinced by the student’s knowledge.	Student’s knowledge is weak, with a lot of room for improvement.	Student has a reasonable grasp of the medical issues, with average knowledge levels.	Student has attained the required level of knowledge	Totally convinced by their level of knowledge. Above and beyond the required level.
Confidence in this student	No confidence in this student. I would never visit this student with a medical problem. Student seems incompetent.	I would be unlikely to visit this student for any medical issue. Student seems below the competency level required.	Medium level confidence in this student. I might be happy to visit this student with a medical problem. Average competence but there is room for improvement.	I am confident that this student has attained the required level. Happy to visit the student with the majority of medical issues. Above average competence level.	I would be perfectly happy to visit this student with any medical problem. I feel like they know exactly what they are doing. I am confident in their ability. They are fully competent.

**Table 8:** Appendix 4: Rubric for Examiners – How to assess students in OSCE domains [30] Rubric used for training Physician Examiners for domain OSCE ratings [30]

	1 – Very Poor	2 – Poor	3 - Average	4 – Good	5 – Very good
Empathy	Little or no empathy. No consideration to the needs of the patient. Self-involved, egocentric. No response to cues or concerns.	Understands that the patient has needs. Little response to patient needs. Limited perspectives. Brushes over patient concerns.	More connected to how the patient sees things and patient perspectives. Makes an attempt to address patient concerns.	Good connection to patient. Better attempt to address patient’s ideas, concerns and expectations. Patient feels his thoughts and feelings are being addressed.	Able to put himself in the patient’s shoes. Addresses patient’s ideas and concerns and actively responds. Patient feels completely at ease. Sympathetic
Communication skills	Closed body language. Closed leading questions. Looking at paper or monitor. No eye contact. No rapport established. Patient not at ease. No information shared. Uses a lot of Medical jargon	Tense body language. Turns slightly to speaker. Minimal eye contact. Mostly closed questions. Concentrated on gathering info. Use of medical jargon	More relaxed. Intermittent eye contact. Turned more towards the patient. Some open questions. No significant response to patient concerns – passive listening.	Mirrors body language of patient. Sustained eye contact. Mixture of open and closed questions. Good rapport. Makes an attempt to share information with the patient. Minimal medical jargon.	Relaxed and open. Natural eye-contact. Listens attentively. Shows interest and respect. Starts with open questions and allows patient to express themselves. Active listening. Picks up cues. Puts patient at complete ease. Shares and checks understanding and management plan. No medical jargon.
Knowledge	Poor knowledge. No grasp of the subject matter. Gives inaccurate information to patient. Formulates no plan or inappropriate unrealistic plan.	Below average knowledge. Inadequate grasp of subject matter. Incomplete or incorrect plan. Little information given to patient.	Average knowledge. Reasonably accurate information. Formulates a reasonable plan. Could give more relevant information and more extensive plan.	Above average knowledge. Shows a good grasp of the subject matter. Offers the patient some relevant information and formulates a reasonable realistic plan.	Excellent knowledge. Knows the subject matter in depth. Gives relevant and accurate information and formulates fully appropriate and realistic plan.
Clinical competence	Student is incompetent, Disorganised consultation. Poor time management. Unable to prioritize. Unsafe. Unfit for internship.	Below average competence. Probably unfit for internship	Average competence. Student borderline for internship.	Above average competence. Student ready for internship.	Fully competent. Student has reached a high level. Organised consultation, professional, excellent time management, prioritizes well. Student well above required level for internship.

Figures 3-5 show the participant information sheet and informed consent forms provided to the simulated patients.
